# Astragaloside IV for Experimental Focal Cerebral Ischemia: Preclinical Evidence and Possible Mechanisms

**DOI:** 10.1155/2017/8424326

**Published:** 2017-02-20

**Authors:** Hui-Lin Wang, Qi-Hui Zhou, Meng-Bei Xu, Xiao-Li Zhou, Guo-Qing Zheng

**Affiliations:** Department of Neurology, the Second Affiliated Hospital and Yuying Children's Hospital of Wenzhou Medical University, Wenzhou, China

## Abstract

Astragaloside IV (AST-IV) is a principal component of Radix Astragali seu Hedysari (Huangqi) and exerts potential neuroprotection in experimental ischemic stroke. Here, we systematically assessed the effectiveness and possible mechanisms of AST-IV for experimental acute ischemic stroke. An electronic search in eight databases was conducted from inception to March 2016. The study quality score was evaluated using the CAMARADES. Rev Man 5.0 software was used for data analyses. Thirteen studies with 244 animals were identified. The study quality score of included studies ranged from 3/10 to 8/10. Eleven studies showed significant effects of AST-IV for ameliorating the neurological function score (*P* < 0.05); seven studies for reducing the infarct volume (*P* < 0.05); and three or two studies for reducing the brain water content and Evans blue leakage (*P* < 0.05), respectively, compared with the control. The mechanisms of AST-IV for ischemic stroke are multiple such as antioxidative/nitration stress reaction, anti-inflammatory, and antiapoptosis. In conclusion, the findings of present study indicated that AST-IV could improve neurological deficits and infarct volume and reduce the blood-brain barrier permeability in experimental cerebral ischemia despite some methodological flaws. Thus, AST-IV exerted a possible neuroprotective effect during the cerebral ischemia/reperfusion injury largely through its antioxidant, anti-inflammatory, and antiapoptosis properties.

## 1. Introduction

**Figure 1:**
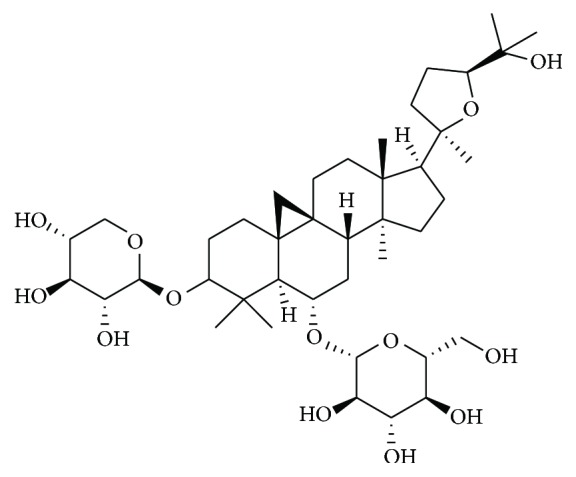
Chemical structures of Astragaloside IV.

Radix Astragali seu Hedysari, milkvetch root (Huangqi), the dried root of* Astragalus membranaceus* (Fisch.) Bge. var. Mongolicus (Bge.) Hsiao or* Astragalus membranaceus* (Fisch.) Bge., is a famous Traditional Chinese Qi-tonifying herb for a numerous disorders [1]. Huangqi was originally described in the* Shennong Bencaojing* (*Shennong's Classic of Materia Medica*), the earliest complete Pharmacopoeia of China written from Warring States Period to Han Dynasty [2]. Specifically, Huangqi has been used to treat stroke in China for thousands of years and elsewhere around the world in recent years. For example, Buyang Huanwu Decoction is a well-known classic herbal prescription for ischemic stroke [3], in which Huangqi is used as a major medicinal herb, that is, the sovereign drug based on traditional Chinese medicine formula theory. The chemical composition of Huangqi mainly includes saponins, polysaccharides, flavonoids, amino acids, and trace elements, and various biological activities have been reported [4]. Currently, more than 200 constituents are being identified [5]. The main compounds are shown in Table 1. Chemical structure of As-IV is 3-O-beta-D-xylopyranosyl-6-O-beta-D-glucopyranosyl-cycloastragenol, a lanolin alcohol-shaped tetracyclic triterpenoid saponin with high polarity, and its molecular formula is C14H68O14 (Figure 1). AST-IV served as a quality-control marker component of Huangqi in the Chinese Pharmacopoeia (2015 version). The previous studies have demonstrated that AST-IV possesses antioxidant, anti-inflammatory, and antiapoptotic effects on focal cerebral ischemia/reperfusion (I/R), cardiovascular disease, pulmonary disease, liver cirrhosis, and renal disease [6]. In the past decades, there have been a growing number of divergent preclinical researches examining the effects of AST-IV on focal cerebral ischemia in animal models [2]. In addition, a systematic review is a type of literature review that synthesizes all the available evidence focusing on a specific question. Systematic review of preclinical studies has been testified as useful in optimizing the design of both clinical and preclinical studies [7]. This type of review can improve the rigor of the conducting and reporting of preclinical research, provide benefits for further preclinical research, and inform clinical trial design. However, no systematic review has yet been conducted to assess the preclinical evidence of AST-IV for ischemic stroke. Here, we carried out a preclinical systematic review to evaluate the effectiveness and mechanisms of AST-IV for ischemic stroke in animal models.

## 2. Methods

### 2.1. Database and Search Strategies

The following databases were searched: PubMed, Embase, Web of science, Chinese Biomedical Literature Database, China National Knowledge Infrastructure, WanFang Database for Chinese Technical Periodicals, and VIP Database. All studies were performed from inception to March 2016. The abstracts of scientific meetings and reference lists of all included studies were manually searched. Our search term included “[isch(a)emic stroke OR cerebral infarct OR Cerebral reperfusion OR Cerebral isch(a)emia OR Cerebral isch(a)emic injury OR middle carotid artery occlusion (MCAO)] AND Astragaloside IV”. All searches were limited to studies on animals.

### 2.2. Eligibility Criteria

We prespecified the eligibility criteria as follows: (1) we included controlled studies of AST-IV for experimental ischemic stroke; (2) the primary outcomes were measured as neurological function score (NFS), infarct volume (IV), and/or blood-brain barrier (BBB) permeability such as Evans blue and/or brain water content (BWC); the second outcome measures were mechanisms of AST-IV for ischemic stroke; (3) animal model of focal cerebral ischemia was induced by temporary middle cerebral artery occlusion (MCAO); (4) AST-IV was used merely in intervention group; (5) control animals received vehicle or no treatment. Prespecified exclusion criteria were as follows: (1) nonfocal cerebral ischemia model such as global, traumatic models, or hypoxic-ischemic models; (2) permanent MCAO; (3) combined use of any other agents; (4) no control group; (5) duplicate publications.

### 2.3. Data Extraction

Two independent authors extracted the following details from included studies: (1) the first author's name and publication year, model of ischemic stroke, and the anesthesia methods for model preparation; (2) the specific information of animals for each study, including animal species, number, sex, and weight; (3) the treatment group's information, including therapeutic drug dosage, method of administration, duration of treatment, and the same information of control group; (4) the outcomes' data of mean value and standard deviation were extracted from each study, including NFS, IV, and/or BBB permeability, and timing for outcome assessments. The data of highest dose was included when the treatment group included various doses of the target drug. The result of the peak time point was included when the data were expressed at different times. Some records' published data were only expressed graphically, we made efforts to contact authors for further information, and when a response was not received, the numerical values were measured from the graphs by using digital ruler software.

### 2.4. Quality Assessment

The study quality score was independently valued with the Collaborative Approach to Meta-Analysis and Review of Animal Data from Experimental Studies (CAMARADES) [21] 10-item quality checklist by two authors. One point was given for each of the following criteria: publication in a peer reviewed journal, control of temperature, random allocation to treatment or control, blinded induction of model, blinded assessment of outcome, use of an anesthetic without intrinsic neurogenesis activity, animal model (aged, diabetic, or hypertensive), performing a sample size calculation, compliance with animal welfare regulations, and a statement of potential conflicts of interest.

### 2.5. Statistical Analysis

NFS, IV, and BBB permeability were considered as continuous data analyzed by using Review Manager (version 5.0). The estimate of the combined effect sizes was calculated by the standardized mean difference (SMD) utilizing the random effects model. *I*^2^ statistic was used to assess heterogeneity. Statistical significance was set at *P* < 0.05, and the 95% confidence intervals (CIs) of all results were calculated.

## 3. Results

### 3.1. Study Selection

**Figure 2:**
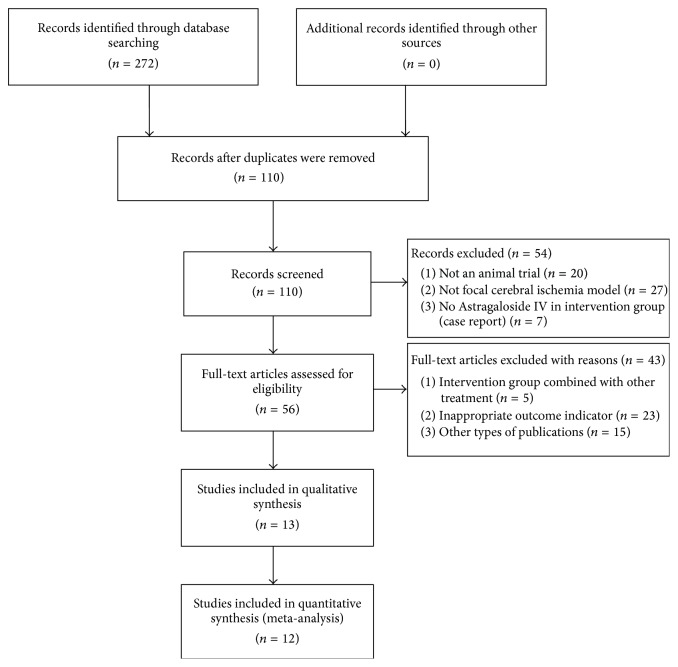
Summary of the process for identifying candidate studies.

We identified 272 potentially relevant articles, of which 162 were duplicates. After screening the titles and abstracts, 54 papers were excluded because they were not (1) animal trials, (2) focal cerebral ischemia model, or (3) AST-IV in intervention group. We then read the remaining 56 full-text articles. Among them, 43 articles were deleted because 5 studies used other combined drugs in experimental group, 23 articles did not possess an appropriate outcome, and the remaining 15 studies were other types of publications. Finally, 13 studies were selected. The screening process is summarized in the flow diagram in Figure 2.

### 3.2. Characteristics of Included Studies

Seven studies [11–13, 16–19] were published in English, and 6 studies [8–10, 14, 15, 20] were published in Chinese between 2000 and 2015. The 13 included studies involved Sprague-Dawley rats [9–11, 13–18, 20], Wistar rats [8, 19], and C57/B6 mice [12]. The weight of rats varied between 230 and 325 g. Twelve studies used male animals and only one study [16] used both female and male mice. To induce anesthesia, 7 studies used chloral hydrate [8, 11–13, 18–20], 2 studies [16, 17] used pentobarbital sodium, 1 study [15] used isoflurane, and the remaining 3 studies did not report anesthetics [9, 10, 14]. The MCAO ischemia time of 12 studies varied from 1 to 2 hours, whereas one study did not mention it [9]. Seven studies implemented the dose gradient of AST-IV. Among them, three studies utilized 10 and 20 mg·kg^−1^ [11, 13, 15], two studies adopted 10, 40, and 100 mg·kg^−1^ [10, 16], one study used 20 and 40 mg·kg^−1^ [12], and the remaining one study [8] applied 1.5, 3, and 6 mg·kg^−1^. Six studies performed single dose, in which two [9, 17] of them used 20 mg·kg^−1^, one [18] did not mention it, and the others [14, 19, 20]used 5, 10, and 40 mg·kg^−1^, respectively. Eight studies [9, 11–14, 17–19] administrated the therapy after ischemia and four studies [10, 15, 16, 20] before ischemia, and one study [8] injected it before ischemia and after reperfusion. The therapy was administrated via intraperitoneal injection in 11 studies [8–14, 16–18, 20] and intragastric in 2 studies [15, 19]. NSF was reported in 11 studies [8–11, 13–19], IV in 7 studies [10–12, 14, 17–19], and BBB in 4 studies [13, 14, 19, 20]. The overall characteristics of included publications are shown in Table 2.

### 3.3. Study Quality

The score of study quality ranged from 3 to 8 in a total of 10 points (Table 3) of which five studies got 3 points [8–10, 14, 20]; four studies got 5 [12, 15, 17, 19]; two studies got 6 [16, 18]; and two studies got 8 [11, 13]. All the included records were peer reviewed publications and random allocations; however, no study reported a sample size calculation and all studies were performed on healthy animals. Control of temperature was described in 8 studies [9–13, 15, 18, 19]. Ten studies used an anesthetic without intrinsic neuroprotective properties [8, 11–13, 15–20]; five reported blinding their assessment of outcome [11, 13, 15, 16, 18]; two reported blinded induction of model [11, 13]; seven reported compliance with animal welfare regulations [11–13, 16–19]; and five studies declared no potential conflict of interests [11, 13, 14, 16, 17].

### 3.4. Effectiveness

**Figure 3:**
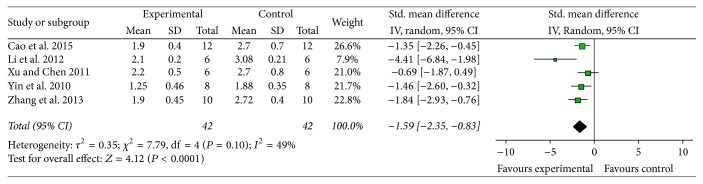
The forest plot: effects of Astragaloside IV for improving the neurological function score compared with middle carotid artery occlusion group according to Bederson criterion.

**Figure 4:**
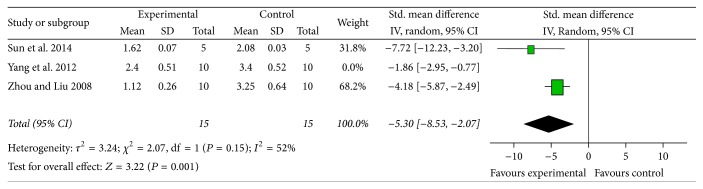
The forest plot: effects of Astragaloside IV for improving the neurological function score compared with middle carotid artery occlusion group according to Longa criterion.

*NFS*. Meta-analysis of 5 studies [10, 11, 14, 16, 19] showed significant effect of AST-IV for improving the NFS compared with control group according to Bederson criterion (*n* = 84, MD −1.59, 95% CI [−2.35, −0.83], *P* < 0.0001; heterogeneity: Tau^2^ = 0.35; Chi^2^ = 7.79, df = 4 (*P* = 0.10); *I*^2^ = 49%) (Figure 3). Meta-analysis of three studies [8, 15, 17] reported significant effect of AST-IV for improving the NFS on Longa criterion (*n* = 50, MD −3.85, 95% CI [−6.40, −1.31], *P* = 0.003; heterogeneity: Tau^2^ = 3.63; Chi^2^ = 9.96, df = 2 (*P* = 0.007); *I*^2^ = 80%). However, owing to obvious heterogeneity, we used sensitivity analyses and removed the respective outlier study. Meta-analysis of two studies [8, 15] indicated that AST-IV significantly improved the NFS compared with control group according to Longa criterion (*n* = 30, MD −5.30, 95% CI [−8.53, −2.07], *P* = 0.001; heterogeneity: Tau^2^ = 3.24; Chi^2^ = 2.07, df = 1 (*P* = 0.15); *I*^2^ = 52%) (Figure 4). In addition, three studies [9, 11, 18] also showed the significant effects of ameliorating the NFS according to the Masuo criteria, Garcia criteria, and 7-point scoring criteria, respectively (*P* < 0.05 or *P* < 0.01).

**Figure 5:**
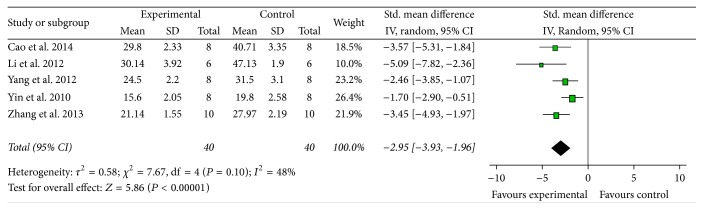
The forest plot: effects of Astragaloside IV for reducing the infarct volume compared with middle carotid artery occlusion group.

*IV*. Eight studies [10–12, 14, 17–19] showed significant effects of AST-IV for reducing the IV or IV% according to the Triphenyltetrazolium chloride (TTC) staining. Meta-analysis of five studies [11, 14, 17–19] showed that AST-IV significantly reduces IV in rat's MCAo model compared with control group (*n* = 80, MD −2.95, 95% CI [−3.93, −1.96], *P* < 0.00001; heterogeneity: Tau^2^ = 0.58; Chi^2^ = 7.67, df = 4 (*P* = 0.10); *I*^2^ = 48%) (Figure 5). Two studies failed for pool analysis because one study used infarct volume as outcome measure [10] and another study used mice as animal model [12]. However, they all got the significant effects for reducing the infarct weight or infarct volume (*P* < 0.05 or *P* < 0.01).

**Figure 6:**
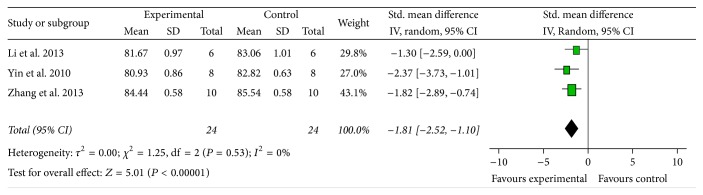
The forest plot: effects of Astragaloside IV for reducing the brain water content compared with middle carotid artery occlusion group.

*BBB*. Both the BWC and the Evans blue were used as outcome measures. Meta-analysis of three studies [13, 14, 19] showed that AST-IV had significant effects for reducing BWC compared with control group (*n* = 48, MD −1.81, 95% CI [−2.52, −1.10], *P* < 0.00001; heterogeneity: Tau^2^ = 0.00; Chi^2^ = 1.25, df = 2 (*P* = 0.53); I^2^ = 0%) (Figure 6). Two studies [13, 20] reported that AST-IV significantly reduced Evans blue extravasation compared with control (*P* < 0.05 or *P* < 0.01).

### 3.5. Mechanisms of AST-IV for Ischemic Stroke

The mechanisms of AST-IV for ischemic stroke are summarized as follows: (1) reduction of oxidative/nitration stress reaction [9–12, 14, 15, 17–19] through decreasing malondialdehyde [12, 14, 15, 17, 19] and nitric oxide [19], and increasing superoxide dismutase [12, 14, 15, 17, 19] and glutathione peroxidase [12, 15]; (2) anti-inflammatory reactions [9–11, 14, 17] through inhibiting the expression of myeloperoxidase [11], tumor necrosis factor-*α* [9, 11], interleukin-1*β* [11], inducible nitric oxide synthase [9, 17, 19], intercellular adhesion molecule-1 [11], and nuclear factor *κ*B (NF-*κ*B) [11], and decreasing the percentage of CD11b/CD18-positive [11]; (3) inhibition of apoptosis [8, 14, 15, 17] through upregulating the expression of B-cell lymphoma/leukemia-2 protein [14, 17] and downregulating the expression of B-cell lymphoma/leukemia-2 associated X protein [14] and caspase-3 [17]; (4) preserving or protecting the integrity of BBB [13, 14, 20]; (5) stimulating the nerve regeneration and promoting the nerve repairmen [8, 19]; (6) promotion of angiogenesis [8]; (7) attenuating the increase of vascular permeability [13]; (8) inhibiting peripheral benzodiazepine receptor expression [16].

## 4. Discussion

### 4.1. Summary of Evidence

Thirteen studies with 244 animals were selected. The quality of many studies included was moderate. Treatment with AST-IV could reduce the IV and BBB and improve NFS outcomes during cerebral ischemia/reperfusion (I/R), suggesting that AST-IV exerted potential neuroprotection in acute ischemic stroke. Mechanisms of AST-IV for neuroprotective effects are largely mediated by its antioxidant, anti-inflammatory, and antiapoptosis properties. Despite the apparent positive results, we should interpret them with caution because of the methodological flaws.

### 4.2. Limitations

First, our analysis only included studies in English and Chinese. This may lead to certain degree to selective bias. Second, negative findings are less likely to be published. Thus, the effect of present study may have been overestimated. Third, high quality of control experimental study has significant impacts on reported outcome. However, many studies were of methodological flaws, suggesting that the weaknesses existed in the primary study.

### 4.3. Implications

Systematic reviews of preclinical animal studies can help improve the methodological quality of animal experiments [22]. Adequate methodological details are crucial to value the quality of a body of evidence and to identify the risk of bias in trials. However, in the present study, the methodology's insufficiency exists in many fields, which potentially leads to an overestimation of treatment effects [23, 24]. In particular, appropriate animal model is one of the important aspects to improve the quality of experimental design. All included studies were performed on healthy animals, in which the conditions of clinical stroke patients with morbidity may not accurately be replicated owning to stroke generally occurring in elders with comorbidities such as hyperglycemia or hypertension [25]. In addition, comorbidities can affect efficacy in animal models [26]. For instance, hypertension might attenuate neuroprotective effects [27]. Thus, there was a need to improve methodological standards in the design, conduct, and reporting of preclinical animal studies in the acute ischemic stroke. Additionally, appropriate animal model with comorbidities such as aging, hyperglycemia, or hypertension should be used in experimental stroke research.

Neuroprotection for ischemic stroke was defined as an innovative strategy for antagonizing the injurious biochemical and molecular events that eventually resulted in irreversible ischemic injury [28]. Although there is identification of over 1000 effective neuroprotectants in animal studies and execution of over 100 clinical trials, successful translation of a neuroprotectant to the routine clinical use for stroke has not yet occurred [29]. Systematic reviews of preclinical stroke studies are useful in identifying the design-related factors such as poor methodological quality, differences in design between animal researches and clinical trials, and publication bias so as to improve the internal and external validity and thereby to increase the predictive value of experimental stroke [22]. Thus, systematic review of preclinical animal studies of stroke can contribute to more evidence-based translation of animal data from the bench to the bedside. The present study showed that AST-IV had potentially neuroprotective effect for acute ischemic stroke in animal models. It provides a preclinical evidence-based approach to the development of new treatments for acute ischemic stroke. Thus, findings of AST-IV from systematic review level in the present study may be collectively used to influence the decision for candidate drugs to be assessed in further clinical trials.

Once the cerebral I/R occurred, two-stage process will be triggered: acute injuries and delayed injuries. Acute injuries are associated with energy metabolism dysfunction, oxidative stress, and the destruction of BBB. During period of I/R, the following inflammatory response and apoptosis exacerbate the injuries [30]. Several signalling pathways of cerebral I/R injury have been studied, including mitogen-activated protein kinase (MAPK) signalling pathways such as c-Jun NH2-terminal kinase 1/2 MAPK and p38 MAPK [31, 32], Toll-like receptors (TLRs) signalling pathways such as TLRs/turn activates activated kinase 1/I*κ*B kinase complex and TLRs/turn activates activated kinase 1/mitogen-activated protein kinase kinase kinase [33], NF-*κ*B signalling pathway [34], phosphatidylinositol 3-kinase/Akt signalling pathway, and extracellular signal-regulated kinase 1/2 signalling pathway [35, 36]. Partially based on the previous publications of mechanisms of AST-IV, it can inhibit apoptosis through the transforming growth factor-*β*1/Smad2 signalling pathway [37], reduce oxidative stress via p38 MAPK pathway [38], diminish the myocardial I/R injury in rats by downregulating the TLR4/NF-*κ*B signalling pathway [39], and stimulate angiogenesis through the phosphatidylinositol 3-kinase/Akt pathway [40]. The present study indicated that AST-IV could attenuate oxidative stress [12, 14, 15, 17–19] and inhibit inflammatory stress [9–11, 14, 17] and apoptosis [8, 14, 15] in acute ischemic stroke of animal models by regulating the downstream signalling cascade molecules, proinflammatory mediators, inflammatory mediators, and antiapoptotic regulator. AST-IV exerted a neuroprotective effect during cerebral I/R injury, largely through its antioxidant, anti-inflammatory, and anti-apoptosis properties. However, the signalling pathways of AST-IV during cerebral I/R were infrequently and incompletely reported. Thus, it is worth exploring this field in the future.

## 5. Conclusion

From a scientific and translational perspective, animal experiment researches should be appropriately designed, well conducted, thoroughly analyzed, and transparently and completely reported. Systematic review of animal studies can help improve the methodological quality of animal experiments and the more evidence-based translation of animal data to the clinic and contribute to the development of replacement, reduction, and refinement of animal experiments. In the present study, the findings demonstrated that AST-IV could improve NFS and IV and reduce the BBB permeability in animal models of focal cerebral ischemia largely through its antioxidant, anti-inflammatory, and antiapoptosis effects. Thus, AST-IV is a potential candidate neuroprotectant in further stroke clinical trials.

## Figures and Tables

**Table 1:** The main compounds isolated from Radix Astragali seu Hedysari (Huangqi).

Category	Main compounds
Saponin	Caspicuside I, Astragalus saponin I–IV, Acetyl Astragalus saponin I, Isoastragaloside IV, Sphondin, Astragaloside I–IV, Astragalus saponins I, Astragalus saponins IV, Astragalus saponins VII, Astragalus aglycone, Baibutoside, Cyclogalegigenin, Saponin-Huangqiyiesaponin C, Mongholicoside A, Mongholicoside B, Tetracyclic triterpenoids, Cotton wool Astragalus saponins, 3-O-*β*-D-Xylopyraosyl-24S-cycloart-3*β*,6*α*,16*β*,24,25-pentaol-25-O-*β*-D-glucopyranoside, Isoastragaloside IV, Cyclocanthoside A, Cyclounifoliside C, Asernestioside C, 6,3′-Dihydroxy-2′,4′-dimethoxyisoflavan-6-O-*β*-Dglucopyranoside, Calycosin-7-O-*β*-D-glucoside, 7,3′-Dihydroxyl-6,4′-dimethoxyisoflavon-7-O-*β*-D-glucopyranoside, *β*-Sitosterol, *β*-Daucosterol
Polysaccharide	*α*-(1→4) (1→6) glucan, *α*-(1→4) glucan
(including glucan and heteropolysaccharide)	That is, glucose, galactose, arabinose, rhamnose, mannose, xylose, fucose, fructose, ribose, glucuronic acid, galacturonic acid
Flavonoid	Kaempferol, Quercetin, Isorhamnetin, Narcissin, Nicotiflorin, Microcephalin I, Microcephalin II, Rhamnocitin, (3R)-2′,3′-Dihydroxy-7,4-dimethyl oxygen radicals isoflavone, Rutin, (6aR,11aR)-10-Hydroxy-3,9-Dimethyl oxygen radicals red sandalwood alkyl, 1′-Hydroxy-7,3′,4,-trimethoxy isoflavone, Odoratin-7-O-*β*-D-glucoside, Salvigenin, Apigenin, Luteolin, 7,3′-Dihydroxy-6,4′-dimethoxyisoflavone, 7-Hydroxyflavone, Formononetin-7-O-*β*-D-glycoside-6′′-O-acetate, (6aR,11aR)-3-Hydroxy-9,10-dimethoxy-pterocarpan, Calycosin-7-O-*β*-D-glucoside-6′′-O-malonate, Formononetin-7-O-*β*-D-glucoside-6′′-D-malonate, (6aR,11aR)-3-Hydroxy-9,10-dimethoxypterocarpan, (3R)-7,2′-Dihydroxy-3′,4′-dimethoxyisoflavan, Astrapterocarpanglucoside-6′-O-malonate, Astraisoflavanglucoside-6′-O-malonate, Calycosin-7-O-*β*-D-glucoside-6′′-O-acetate
Amino acid	Asparagine, canavanine, proline, arginine, aspartic acid, alanine
trace element	scandium, chromium, cobalt, copper, selenium, molybdenum, cesium, iron, manganese, zinc, rubidium
Other compounds	Coumarin, folic acid, bitter elements, choline, betaine, linoleic acid, linolenic acid, vanillic acid, ferulic acid, isoferulic acid, paimitie acid, hydroxy phenyl acrylic acid, caffeic acid, green acid, palm acid, 13-sitosterol, daucosterol, lupeol

**Table 2:** Characteristics of the 13 included studies.

Study (years)	Species (sex, *n* = experimental/control group)	Weight	Model (method)	Anesthetic	Treatment group (Astragaloside IV)	Control group	Outcome index (time)	Intergroup differences
Sun et al. 2014 [8]	Wistar rats (male, 5/5)	230~250 g	MACO IR/2 H	10% chloral hydrate (3 mL/kg)/(intraperitoneal injection)	1.5, 3, 6 mL/kg (1 mg/mL AST-IV), ip At (1) 0.5 H before ischemia (2) 0 H after reperfusion (3) 24 H after reperfusion (4) 48 H after reperfusion	Model group 6 mL/kg, ip, 0.9% NS At (1) 0.5 H before ischemia (2) 0 H after reperfusion (3) 24 H after reperfusion (4) 48 H after reperfusion	(1) NFS (Longa) (72 h after reperfusion) (2) morphological structure of cortical neurons (3) apoptosis (4) BDNF (5) VEGF (6) VEGFR2	(1) *P* < 0.05 (2) */* (3) *P* < 0.05 (4) *P* < 0.01 (5) *P* < 0.05 (6) *P* < 0.05

Huang et al. 2013 [9]	SD rats (male, 6/6)	About 250 g	MACO IR	NE	20 mg/kg, ip At 0 H after reperfusion, then each 4 H after reperfusion until 24 H	Model group 5 mL/kg, ip, 0.9% NS At 0 H after reperfusion, then each 4 H after reperfusion until 24 H	(1) NFS (7-point) (waking after reperfusion) (2) TNF-*α* (3) the mRNA of iNOS	(1) *P* < 0.05 (2) *P* < 0.05 (3) *P* < 0.01

Xu and Chen 2011 [10]	SD rats (male, 6/6)	230~250 g	MACO IR/2 H	NE	10, 40, 100 mg/kg, ip At 0.5 H before ischemia	Model group The same volume of 0.9% NS compared with treatment group, ip At (1) 0.5 H before ischemia	(1) NFS (Bederson) (24 h after reperfusion) (2) IV (TTC) (24 h after reperfusion) (3) GFAP	(1) *P* < 0.05 (2) *P* < 0.05 (3) *P* < 0.05

Li et al. 2012 [11]	SD rats (male, 6/6)	250–320 g	MACO IR/1.5 H	5% chloral hydrate (400 mg/kg)/(intraperitoneal injection)	10, 20 mg/kg, ip At (1) 0 H after reperfusion (2) 12 H after reperfusion	Model group The same volume of 0.9% NS compared with treatment group, ip At (1) 0 H after reperfusion (2) 12 H after reperfusion	(1) NFS (Garcia 18-point) (24 h after reperfusion) (2) IV (TTC) (24 h after reperfusion) (3) MPO concentration (4) the upregulation of IL-1*β* and TNF-*α* (5) CD11b/CD18-positive percentage of neutrophils (6) the expression of ICAM-1 (7) the expression of NF-*κ*B	(1) *P* < 0.05 (2) *P* < 0.05 (3) *P* < 0.05 (4) *P* < 0.05 (5) *P* < 0.05 (6) *P* < 0.05 (7) *P* < 0.05

Luo et al. 2004 [12]	C57/B6 mice (male, 15/15)	25–30 g	MACO IR/1.5 H	chloral hydrate (300 mg/kg)/(intraperitoneal injection)	20, 40 mg/kg, ip At (1) 0 H after ischemia (2) 24 H after reperfusion (3) 48 H after reperfusion	Model group The same volume of saline compared with treatment group At (1) 0 H after ischemia (2) 24 H after reperfusion (3) 48 H after reperfusion	(1) IV (TTC) (72 h after operation) (2) MDA (3) SOD (4) GSH-PX	(1) *P* < 0.03 (2) *P* < 0.01 (3) *P* < 0.01 (4) *P* < 0.01

Li et al. 2013 [13]	SD rats (male, 6/6)	250–320 g	MACO IR/1.5 H	5% chloral hydrate (400 mg/kg)/(intraperitoneal injection)	10, 20 mg/kg, ip At (1) 0 H after reperfusion (2) 12 H after reperfusion	Model group The same volume of 0.9% NS compared with treatment group, ip At (1) 0 H after reperfusion (2) 12 H after reperfusion	(1) NFS (Bederson) (24 h after reperfusion) (2) BBB (brain water content) (NR) (3) BBB (Evans blue) (24 h after reperfusion) (4) vascular permeability (5) immunohistochemical analysis of MMP-9 and AQP4	(1) *P* < 0.05 (2) *P* < 0.05 (3) *P* < 0.05 (4) */* (5) *P* < 0.05

Zhang et al. 2013 [14]	SD rats (male, 10/10)	280 ± 10 g	MACO IR/1 H	NE	5 mg/kg, ip At (1) 0 H after reperfusion (2) 6 H after reperfusion (3) 23 H after reperfusion	Model group The same volume of 0.9% NS compared with treatment group At (1) 0 H after reperfusion (2) 6 H after reperfusion (3) 23 H after reperfusion	(1) NFS (Bederson) (24 h after reperfusion) (2) IV (TTC) (24 h after reperfusion) (3) BBB (brain water content) (24 h after reperfusion) (4) Brain index (5) Activity of SOD (6) Content of MDA (7) Bcl-2 protein (8) Bax expression (9) Reducing or disappearing of Nissl body	(1) *P* < 0.05 (2) *P* < 0.01 (3) *P* < 0.05 (4) *P* < 0.01 (5) *P* < 0.01 (6) *P* < 0.05 (7) *P* < 0.05 (8) *P* < 0.01 (9) *P* < 0.05

Zhou and Liu 2008 [15]	SD rats (male, 10/10)	280–320 g	MACO IR/2 H	40 mL/L isoflurane induction then 20 mL/L isoflurane inhaled maintenance anesthesia	10, 20 mg/kg, intragastric At (1) 3 D before reperfusion (2) 2 D before reperfusion (3) 1 H before reperfusion	Model group The same volume of 0.9% NS compared with treatment group At (1) 3 D before reperfusion (2) 2 D before reperfusion (3) 1 H before reperfusion	(1) NFS (Longa) (24 h after reperfusion) (2) Infarct weight (TTC) (24 h after reperfusion) (3) SOD (4) GSH-PX (5) MDA (6) Apoptosis index	(1) *P* < 0.01 (2) *P* < 0.01 (3) *P* < 0.01 (4) *P* < 0.01 (5) *P* < 0.01 (6) *P* < 0.01

Cao et al. 2015 [16]	SD rats (6 males and 6 females, 12/12)	250–300 g	MACO IR/2 H	2% pentobarbital sodium (40 mg/kg)/(intraperitoneal injection)	10, 40, 100 mg/kg, ip At 0.5 H before reperfusion	Model group The same volume of 0.9% NS compared with treatment group At 0.5 H before reperfusion	(1) NFS (Bederson) (24 h after reperfusion) (2) Mitochondrial [^3^H]PK11195 binding	(1) *P* < 0.01 (2) *P* < 0.05

Yang et al. 2012 [17]	SD rats (male, 10/10 (8/8))	280 ± 20 g	MACO IR/1 H	1% pentobarbital sodium (40 mg/kg)/(intraperitoneal injection)	20 mg/kg, ip At 0 H, 12 H, 1 D, 2 D, 3 D, till 7 D after reperfusion	Model group The same dose of sterile saline compared with treatment group At 0 H, 12 H, 1 D, 2 D, 3 D, till 7 D after reperfusion	(1) NFS (Longa) (24 h after reperfusion) (2) IV (TTC) (24 h after reperfusion) (3) Brain glucose metabolism (4) SOD (5) MDA (6) iNOS (7) Caspase-3 (8) Bcl-2	(1) *P* < 0.01 (2) *P* < 0.01 (3) *P* < 0.05 (4) *P* < 0.01 (5) *P* < 0.01 (6) *P* < 0.01 (7) *P* < 0.05 (8) *P* < 0.05

Cao et al. 2014 [18]	SD rats (male, 8/8)	280 ± 20 g	MACO IR/2 H	chloral hydrate (400 mg/kg)/(intraperitoneal injection)	3 mL/kg, ip At 0 H after reperfusion	Model group The same volume of normal saline compared with treatment group At 0 H after reperfusion	(1) NFS (Masuo) (24 h after reperfusion) (2) IV (TTC) (24 h after reperfusion) (3) WBV, PV (4) ROS (5) SOD, GSH-PX, MDA (6) Nrf2	(1) *P* < 0.05 (2) *P* < 0.05 (3) */* (4) *P* < 0.05 (5) */* (6) */*

Yin et al. 2010 [19]	Wistar rats (male, 8/8)	250–325 g	MACO IR/2 H	10% chloral hydrate (300 mg/kg)/(intraperitoneal injection)	40 mg/kg, intragastric At reperfusion 0 H, 8 H, 24 H, then once a day until 14 D	Model group The same time with treatment group	(1) NFS (Bederson) (3 D after reperfusion) (2) IV (TTC) (14 D after reperfusion) (3) BBB (brain water content)/(at 3 D after reperfusion) (4) MDA (7 D) (5) SOD (7 D) (6) NO, NOS (7 D) (7) LD (7 D) (8) LDH (7 D) (9) iNOS (7 D), NGF (7 D), TrkA mRNA (14 D)	(1) *P* < 0.01 (2) *P* < 0.01 (3) *P* < 0.01 (4) *P* < 0.05 (5) *P* < 0.01 (6) *P* < 0.05 (7) *P* < 0.05 (8) *P* < 0.01 (9) *P* < 0.05

Luo and Qin 2000 [20]	SD rats (male, 20/20)	260 ± 10 g	MACO IR/1 H	10% chloral hydrate (3 mL/kg)/(intraperitoneal injection)	10 mg/kg, ip At 1 H before reperfusion	Model group the same volume of normal saline compared with treatment group At 1 H before reperfusion	BBB (Evans blue) (23 h after reperfusion)	*P* < 0.01

*Note*. SD rats: Sprague-Dawley rats. NR: no report. MACO: middle carotid artery occlusion. IR: ischemic reperfusion. Ip: intraperitoneal injection. H: hour. D: day. AST-IV: Astragaloside IV. NS: normal saline. NFS: neurological function score. IV: infarct volume. TTC: triphenyltetrazolium chloride. BBB: blood-brain barrier. BDNF: brain derived neurotrophic factor. VEGF: vascular endothelial growth factor. VEGFR2: receptor of vascular endothelial growth factor. GFAP: glial fibrillary acidic protein. MPO: myeloperoxidase. TNF-*α*: tumor necrosis factor-*α*. iNOS: inducible nitric oxide synthase. NOS: nitric oxide synthase. IL-1*β*: interleukin-1*β*. ICAM-1: intercellular adhesion molecule-1. NF-*κ*B: nuclear factor *κ*B. MDA: malondialdehyde. SOD: superoxide dismutase. GSH-PX: glutathione peroxidase. MMP-9: matrix metalloproteinase-9. AQP4: aquaporin 4. Bcl-2: B-cell lymphoma/leukemia-2. Bax: B-cell lymphoma/leukemia-2 associated X protein. WBV: whole blood viscosity. PV: plasma viscosity. ROS: reactive oxygen species. Nrf2: nuclear factor erythroid 2-related factor 2. LD: lactate. LDH: lactate dehydrogenase. NGF: nerve growth factor. TrkA: tropomyosin receptor kinase A. NO: nitric oxide.

**Table 3:** Risk of bias of the included studies.

Study	A	B	C	D	E	F	G	H	I	J	Total
Sun et al. 2014 [8]	√		√			√					3
Huang et al. 2013 [9]	√	√	√								3
Xu and Chen 2011 [10]	√	√	√								3
Li et al. 2012 [11]	√	√	√	√	√	√			√	√	8
Luo et al. 2004 [12]	√	√	√			√			√		5
Li et al. 2013 [13]	√	√	√	√	√	√			√	√	8
Zhang et al. 2013 [14]	√		√							√	3
Zhou and Liu 2008 [15]	√	√	√		√	√					5
Cao et al. 2015 [16]	√		√		√	√			√	√	6
Yang et al. 2012 [17]	√		√			√			√	√	5
Cao et al. 2014 [18]	√	√	√		√	√			√		6
Yin et al. 2010 [19]	√	√	√			√			√		5
Luo and Qin 2000 [20]	√		√			√					3

*Note*. Studies fulfilling the criteria of A: peer reviewed publication; B: control of temperature; C: random allocation to treatment or control; D: blinded induction of model; E: blinded assessment of outcome; F: use of anesthetic without significant intrinsic neuroprotective activity; G: animal model (aged, diabetic, or hypertensive); H: sample size calculation; I: compliance with animal welfare regulations; J: statement of potential conflict of interests.
